# Oestrogen replacement fails to fully revert ovariectomy-induced changes in adipose tissue monoglycerides, diglycerides and cholesteryl esters of rats fed a lard-enriched diet

**DOI:** 10.1038/s41598-021-82837-6

**Published:** 2021-02-15

**Authors:** Valter Tadeu Boldarine, Ellen Joyce, Amanda Paula Pedroso, Mônica Marques Telles, Lila Missae Oyama, Allain Amador Bueno, Eliane Beraldi Ribeiro

**Affiliations:** 1 Departamento de Fisiologia, Escola Paulista de Medicina, Universidade Federal de São Paulo, Rua Botucatu 862, 2º andar, Vila Clementino, São Paulo, SP 04023-062 Brasil; 2 Department of Biological Sciences, College of Health, Life and Environmental Sciences, University of Worcester, Worcester, UK

**Keywords:** Physiology, Endocrinology

## Abstract

Menopause may be accompanied by abdominal obesity and inflammation, conditions accentuated by high-fat intake, especially of saturated fat (SFA)-rich diets. We investigated the consequences of high-SFA intake on the fatty acid (FA) profile of monoglycerides, diglycerides and cholesteryl esters from retroperitoneal white adipose tissue (RET) of rats with ovariectomy-induced menopause, and the effect of oestradiol replacement. Wistar rats were either ovariectomized (Ovx) or sham operated (Sham) and fed either standard chow (C) or lard-enriched diet (L) for 12 weeks. Half of the Ovx rats received 17β-oestradiol replacement (Ovx + E2). Body weight and food intake were measured weekly. RET neutral lipids were chromatographically separated and FAs analysed by gas chromatography. Ovariectomy alone increased body weight, feed efficiency, RET mass, leptin and insulin levels, leptin/adiponectin ratio, HOMA-IR and HOMA-β indexes. OvxC + E2 showed attenuation in nearly all blood markers. HOMA-β index was restored in OvxL + E2. OvxC showed significantly disturbed SFA and polyunsaturated FA (PUFA) profile in RET cholesteryl esters (CE). OvxC also showed increased monounsaturated FA (MUFA) in the monoglyceride diglyceride (Mono–Di) fraction. Similar changes were not observed in OvxL, although increased SFA and decreased PUFA was observed in Mono–Di. Overall, HRT was only partially able to revert changes induced by ovariectomy. There appears to be increased mobilization of essential FA in Ovx via CE, which is a dynamic lipid species. The same results were not found in Mono–Di, which are more inert. HRT may be helpful to preserve FA profile in visceral fat, but possibly not wholly sufficient in reverting the metabolic effects induced by menopause.

## Introduction

Menopause is a period in women’s lives mainly characterized by the loss of ovarian hormones, during which the risk of developing central obesity is higher than in any other period^1^. Menopause is associated with greater risk of other metabolic occurrences, including weight gain, and the lack of oestrogens after menopause is associated with a shift of fat distribution from subcutaneous to visceral depots, with impaired sensitivity of insulin and leptin, and subsequent predisposition to diabetes^2,3^.

Experimentally, rodent bilateral ovariectomy has been used as a useful model for the investigation of metabolic effects induced by the lack of oestrogen^4,5^. Our group has previously demonstrated that the intake of a lard-enriched high-fat diet by ovariectomized rats potentiated gains in body adiposity and induced metabolic alterations^6^. Saturated fat intake has been shown to induce a proinflammatory state mainly in visceral adipose tissue. In obese postmenopausal women, high saturated fat intake induced fatty acid (FA) accumulation, particularly palmitic acid^7,8^. Diets rich in polyunsaturated fatty acids (PUFA) are also known to induce dramatic metabolic changes; while n-6 PUFA enriched-diets are known to increase pro-inflammatory lipid biomarkers, n-3 PUFA enriched-diets are commonly associated with anti-inflammatory or inflammation-supressing properties^9^.

The primary function of adipocytes, the predominant cell type in white adipose tissue, is energy storage. Adipocytes also present remarkable secretory activity, through the release of a broad range of adipokines and cytokines that influence autocrine, paracrine and endocrine actions on energy metabolism, homeostasis and substrate utilization within adipose, liver and skeletal muscle tissues as well as the brain^10–15^.

Cholesterol is an important structural component of adipocyte membranes, interacting with several steroid hormones that are produced not only in adipose tissue^16,17^, but in other distal tissues including the adrenal and sex glands^18,19^. Cholesterol exerts its function by preventing sterol response element-binding proteins acting as transcription factors of enzymes in the cholesterol biosynthetic pathway, thereby inhibiting sterol biosynthesis in a classical feedback mechanism^20^.

Neutral lipids, mainly cholesteryl esters, triglycerides, diglycerides, monoglycerides and free fatty acids, are uncharged hydrophobic molecules that play a major role in energy storage^21,22^. Cholesteryl esters (CE) are formed through ester bonds between the carboxyl group of a FA and the hydroxyl group of the cholesterol molecule, with the FA composition of CE being a direct reflection of nutritional status^23–25^.

Given that cholesterol is found abundantly in lipid rafts within the plasma membrane, and considering its role in sterol biosynthesis in organs such as the liver, intestines and gonads^26,27^, the influence of FA composition upon CE availability and oestrogen metabolism becomes a matter of further exploration.

Triglycerides can be conversely dismantled and reassembled to monoglycerides and diglycerides (Mono–Di) depending upon specific metabolic purposes^28^. Furthermore, intracellular neutral lipids store essential fatty acids (EFA) during periods of abundant EFA intake, buffering EFA blood levels. Depending on physiological circumstances, EFA are mobilized for the synthesis of pro- or anti-inflammatory lipid mediators, which modulate a range of immuno-metabolic signalling pathways^29^.

Given the detrimental consequences of metabolic disorders associated with ovarian failure and the chronic intake of energy dense diets upon quality of life of affected individuals, expanding the current knowledge on visceral adipose tissue metabolism paves the way for future, more efficient clinical and dietetic interventions. Our group has recently demonstrated that ovariectomy in control diet-fed rats favoured a disruption of the fatty acid composition of total lipid extract (which includes both polar and non-polar lipids, and most likely reflects the composition of triglycerides) obtained from retroperitoneal white adipose tissue (RET), evidencing a direct disturbance of ovariectomy upon lipid metabolism^30^. Such findings suggest a greater than initially thought role of fatty acid composition in the development of obesity, along with the appearance of an inflammatory state, in that condition. As CE, monoglycerides and diglycerides are neutral lipid species in dynamic structural interchangeability within the white adipose tissue, the present study examined in rats the effects of ovariectomy and lard-enriched diet intake, associated or not with oestrogen replacement therapy, on the RET FA profile of CE and Mono–Di lipid fractions.

## Results

### High-fat diet intake further exacerbates the deleterious effects of ovariectomy

As shown in Table 1, the success of ovariectomy was confirmed by the lower uterus weight in the Ovx groups. Body weight at the beginning of the study was similar amongst the six groups, whilst the four ovariectomized groups (OvxC, OvxC + E2, OvxL, OvxL + E2) showed increased body weight, feed efficiency and RET fat mass at the end of the 12 weeks period. OvxL and OvxL + E2 showed further increased body weight and RET mass in comparison to OvxC and OvxC + E2. Ovariectomy alone or in combination with the high-fat diet significantly increased leptin and insulin levels, leptin/adiponectin ratio, HOMA-IR and HOMA-β indexes. Oestradiol replacement therapy was able to attenuate the effects of ovariectomy in the control diet group while only restoring the HOMA-β index in the lard-fed group. Total cholesterol, HDL-cholesterol and triglycerides levels did not differ significantly amongst the groups.

**Table 1:** Body and serum parameters of ovariectomized (Ovx) or Sham operated rats, fed a control (C) or lard-enriched (L) diet, subjected or not to oestrogen replacement therapy (E2).

	ShamC	OvxC	OvxC + E2	ShamL	OvxL	OvxL + E2
Initial body weight (g)	264.7 ± 5.2	263.0 ± 3.7	263.5 ± 5.0	266.6 ± 4.2	272.8 ± 3.7	261.5 ± 4.3
Final body weight (g)	280.8 (272.3–285.7)	338.0 (336.0–339.5)*	335.7 (325.8–337.0)*	326.3 (307.1–343.4)	410.2 (377.0–418.1)*^#$^	381.3 (357.2–418.1)*^&$^
Feed efficiency (g/Kcal)	2.7 ± 0.5	13.5 ± 0.6*	11.9 ± 0.8*	5.8 ± 1.0	17.3 ± 0.8*^$^	15.2 ± 1.8*^$^
Uterus (g)	0.4 ± 0.0	0.1 ± 0.0*	0.2 ± 0.0*	0.5 ± 0.1	0.1 ± 0.1*	0.2 ± 0.1*
RET mass (g/100 g)	1.1 ± 0.1	2.0 ± 0.2*	1.7 ± 0.1*	2.2 ± 0.1*	2.8 ± 0.2*^#^	2.7 ± 0.2*^&^
Leptin (ng/mL)	2.1 (1.8–2.6)	10.0 (7.8–12.5)*	5.9 (4.4–9.8)	5.2 (2.6–8.9)	12.5 (9.7–13.5)*^$^	13.8 (10.4–14.6)*^$^
Adiponectin (µg/mL)	5.6 (4.3–7.1)	8.0 (7.2–9.3)	7.6 (6.8–8.5)	5.8 (4.9–6.9)	7.1 (5.9–8.1)	6.2 (4.9–7.2)
Leptin/Adiponectin	0.4 (0.2–0.5)	1.4 (1.0–1.9)*	0.8 (0.6–0.9)	0.9 (0.8–1.1)	1.6 (1.4–1.8)*^$^	1.8 (1.6–2.0)*^&$^
Glucose (mg/dL)	92.8 ± 2.9	105.7 ± 6.3	103.8 ± 6.2	107.2 ± 3.9	110.3 ± 6.3*	120.4 ± 5.2*^&^
Insulin (ng/mL)	0.5 ± 0.0	2.3 ± 0.20*	1.3 ± 0.1	1.4 ± 0.1	2.1 ± 0.2*	2.2 ± 0.1*
HOMA-IR	3.1 (2.3–4.2)	14.37(12.0–19.0)*	7.9 (6.2–8.2)	9.4 (7.5–11.6)	16.3 (12.0–19.0)*	18.4 (6.9–22.7)*^&$^
HOMA-β	0.1 (0.1–0.2)	0.5 (0.3–0.7)*	0.2 (0.2–0.4)	0.2 (0.1–0.3)	0.5 (0.2–0.6)*	0.3 (0.2–0.4)
Total cholesterol (mg/dL)	115.4 (104.9–121.7)	140.8 (119.0–193.1)	126.3 (119.8–133.9)	102.4 (92.5–106.2)	99.5 (95.4–112.7)	110.5 (91.2–121.2)
HDL cholesterol (mg/dL)	205.7 (179.0–251.9)	163.1 (146.1–174.0)	142.3 (135.5–157.6)	190.8 (179.1–215.4)	152.3 (145.1–184.0)	149.3 (125.5–156.6)
Triglycerides (mg/dL)	99.1 (94.6–108.2)	111.2 (105.3–118.8)	122.5 (107.7–220.4)	88.61 (80.7–113.4)	105.6 (86.7–115.8)	98.8 (91.6–117.6)

Data presented as mean ± standard error (SE) for variables with normal distribution or median-interquartile range (Q1-Q3) for variables not normally distributed. n = 6 animals per group. * p < 0.05 vs. ShamC;# p < 0.05 OvxC vs.OvxL;& p < 0.05 OvxC + E2 vs.OvxL + E2; $ p < 0.05 vs.ShamL.

### Ovariectomy modifies RET monoglyceride, diglyceride and cholesteryl ester FA composition, which is marginally restored by oestrogen replacement therapy

The FA composition of RET CE and Mono–Di fractions from rats fed the control diet are presented in Tables 2 and 3. Regarding the CE fraction (Table 2), the sum of SFA (∑SFA) was significantly decreased in OvxC in comparison to ShamC, mainly as a consequence of the significantly decreased C16:0 and C18:0. OvxC + E2 showed partial restoration of the ∑SFA, but which did not reach statistical significance in relation to OvxC. The C18/C18:1 ratio was decreased in OvxC compared to ShamC, but this statistically significant difference disappeared when comparing OvxC + E2 *versus* ShamC. The sum of monounsaturated fatty acids (∑MUFA) was statistically similar amongst ShamC, OvxC and OvxC + E2.

**Table 2:** Fatty acid composition of RET cholesteryl ester lipids obtained from ovariectomized (Ovx) or Sham operated rats, fed a control (C) diet, subjected or not to oestrogen replacement therapy (E2).

Fatty acid		% of total fatty acids		
		ShamC	OvxC	OvxC + E2
C14:0	Myristic acid	0.59 ± 0.15	0.59 ± 0.11	0.63 ± 0.14
C16:0	Palmitic acid	22.68 ± 2.42	14.92 ± 2.29*	18.46 ± 2.19*
C18:0	Stearic acid	7.02 ± 1.45	3.20 ± 1.04*	4.04 ± 1.34*
C20:0	Arachidic acid	0.12 ± 0.01	0.01 ± 0.01	0.05 ± 0.01
C24:0	Behenic acid	0.08 ± 0.02	0.44 ± 0.13*	0.21 ± 0.25
∑ SFA		30.37 ± 3.10	19.15 ± 3.29*	23.39 ± 1.84*
C14:1n-7	Myristoleic acid	0.03 ± 0.01	0.04 ± 0.01	0.02 ± 0.01
C16:1n-7	Palmitoleic acid	1.91 ± 0.39	2.03 ± 0.70	1.72 ± 0.60
C18:1n-9	Oleic acid	24.02 ± 4.58	21.65 ± 4.43	25.98 ± 6.42
C18:1n-7	cis-vaccenic acid	2.21 ± 0.63	1.82 ± 0.24	2.17 ± 0.26
C20:1n-9	Eicosenoic acid	0.16 ± 0.10	0.10 ± 0.02	0.11 ± 0.05
C18/C18:1		0.29 ± 0.13	0.13 ± 0.02*	0.15 ± 0.07
C16:0/C16:1n-7		12.21 ± 2.46	7.73 ± 1.68	11.72 ± 4.0
∑ MUFA		28.35 ± 4.93	25.64 ± 4.92	30.01 ± 6.50
C18:3n-3	Alpha-linolenic acid (ALA)	1.06 ± 0.44	5.36 ± 1.22*	3.19 ± 1.64*^#^
C20:5n-3	Eicosapentaenoic acid (EPA)	0.01 ± 0.02	0.15 ± 0.05*	0.10 ± 0.07
C22:5n-3	Docosapentaenoic acid (DPA)	0.11 ± 0.01	0.32 ± 0.15*	0.20 ± 0.11
C22:6n-3	Docosahexaenoic acid (DHA)	0.08 ± 0.02	0.52 ± 0.26*	0.28 ± 0.20
∑ n-3		1.22 ± 0.43	6.36 ± 1.51*	3.76 ± 1.95*^#^
C18:2n-6	Linoleic acid (LA)	27.07 ± 5.15	44.38 ± 6.08*	40.09 ± 5.52*
C18:3n-6	Gamma-linoleic acid	0.01 ± 0.01	0.24 ± 0.07	0.12 ± 0.04
C20:2n-6	Eicosadienoic acid (EDA)	0.19 ± 0.05	0.18 ± 0.03	0.24 ± 0.11
C20:3n-6	Dihomo-gamma linoleic acid	0.56 ± 0.11	0.39 ± 0.09	0.30 ± 0.16*
C20:4n-6	Arachidonic acid (AA)	0.41 ± 0.12	1.69 ± 0.58*	1.10 ± 0.50
C22:4n-6	Docosatetraenoic acid (DTA)	0.08 ± 0.05	0.31 ± 0.15*	0.21 ± 0.05
∑ n-6		28.29 ± 5.20	47.12 ± 6.73*	42.06 ± 6.05*
∑ n-6/∑ n-3		24.31 ± 4.97	7.58 ± 1.04*	13.32 ± 5.72*
∑ PUFA		29.51 ± 2.4	53.75 ± 3.2*	45.82 ± 2.5*
∑ UFA		57.86 ± 9.22	79.11 ± 3.28*	75.83 ± 1.77*
∑ SFA/∑ UFA		0.54 ± 0.14	0.24 ± 0.05*	0.31 ± 0.03*

Data presented as means ± standard error (SE) of the % of total FAs. n = 6 for each group.

*SFA* saturated fatty acids, *MUFA* monounsaturated fatty acids, *PUFA* polyunsaturated fatty acids.

*p < 0.05 vs ShamC; #p < 0.05 OvxC vs OvxC + E2.

**Table 3:** Fatty acid composition of RET monoglyceride diglyceride lipids obtained from ovariectomized (Ovx) or Sham operated rats, fed a control (C) diet, subjected or not to oestrogen replacement therapy (E2).

Fatty acid		% of total fatty acids		
		ShamC	OvxC	OvxC + E2
C14:0	Myristic acid	0.55 ± 0.24	0.85 ± 0.21	0.84 ± 0.46
C16:0	Palmitic acid	21.52 ± 2.44	22.29 ± 1.13	24.62 ± 2.35
C18:0	Stearic acid	5.82 ± 3.56	5.55 ± 1.10	6.36 ± 3.14
C20:0	Arachidic acid	0.08 ± 0.06	0.05 ± 0.01	0.05 ± 0.03
∑ SFA		28.63 ± 3.38	29.17 ± 1.03	32.36 ± 4.99
C14:1n-7	Myristoleic acid	0.01 ± 0.01	0.02 ± 0.01	0.02 ± 0.02
C16:1n-7	Palmitoleic acid	2.01 ± 0.46	2.23 ± 0.75	1.66 ± 0.60
C18:1n-9	Oleic acid	24.12 ± 4.14	32.55 ± 5.55*	27.50 ± 2.38
C18:1n-7	cis-vaccenic acid	2.12 ± 0.23	2.30 ± 0.29	2.44 ± 0.28
C20:1n-9	Eicosenoic acid	0.11 ± 0.02	0.20 ± 0.05*	0.13 ± 0.03^#^
C18:0/C18:1		0.25 ± 0.23	0.16 ± 0.03	0.22 ± 0.11
C16:0/C16:1n-7		11.13 ± 2.58	10.64 ± 2.52	16.25 ± 5.65
∑ MUFA		29.02 ± 3.03	36.82 ± 5.47*	32.02 ± 2.65
C18:3n-3	Alpha-linolenic acid (ALA)	1.58 ± 0.43	1.36 ± 0.45	1.39 ± 0.13
C20:5n-3	Eicosapentaenoic acid (EPA)	0.04 ± 0.02	0.05 ± 0.05	0.03 ± 0.02
C22:5n-3	Docosapentaenoic acid (DPA)	0.08 ± 0.03	0.02 ± 0.03*	0.06 ± 0.01^#^
C22:6n-3	Docosahexaenoic acid (DHA)	0.11 ± 0.04	0.05 ± 0.03*	0.07 ± 0.03
∑ n-3		1.82 ± 0.46	1.48 ± 0.54	1.55 ± 0.17
C18:2n-6	Linoleic acid (LA)	33.02 ± 6.70	28.41 ± 3.02	30.29 ± 5.01
C18:3n-6	Gamma-linoleic acid	0.03 ± 0.02	0.02 ± 0.03	0.03 ± 0.02
C20:2n-6	Eicosadienoic acid (EDA)	0.18 ± 0.02	0.19 ± 0.01	0.16 ± 0.03
C20:3n-6	Dihomo-gamma linoleic acid	0.45 ± 0.11	0.30 ± 0.20	0.27 ± 0.05
C20:4n-6	Arachidonic acid (AA)	1.21 ± 1.23	0.57 ± 0.31	0.57 ± 0.14
C22:4n-6	Docosatetraenoic acid (DTA)	0.17 ± 0.09	0.10 ± 0.08	0.09 ± 0.04
∑ n-6		35.05 ± 5.67	29.59 ± 3.55	31.40 ± 5.15
∑ n-6/∑ n-3		20.07 ± 4.41	21.57 ± 5.93	20.26 ± 3.19
∑ PUFA		36.87 ± 2.4	31.07 ± 3.2	32.95 ± 2.5
∑ UFA		65.89 ± 7.70	67.90 ± 1.78	64.97 ± 6.09
∑ SFA/∑ UFA		0.45 ± 0.11	0.43 ± 0.02	0.51 ± 0.13

Data presented as means ± standard error (SE) of the % of total FAs. n = 6 for each group.

*SFA* saturated fatty acids, *MUFA* monounsaturated fatty acids, *PUFA* polyunsaturated fatty acids.

*p < 0.05 vs ShamC; #p < 0.05 OvxC vs OvxC + E2.

OvxC rats showed increased ∑n-3, ∑n-6 and ∑PUFA when compared to ShamC. OvxC + E2 showed a partial restoration of this change, being significantly lower than OvxC in ∑n-3, but not reaching significant differences in ∑n-6 and ∑PUFA. This phenomenon is particularly noticeable across the truly essential fatty acids alpha-linolenic acid (C18:3n-3) and linolenic acid (C18:2n-6). C18:3n-3 is higher in OvxC as compared to ShamC, whilst OvxC + E2 is found at the mid-range, being significantly different from both OvxC and ShamC. C18:2n-6 is higher in OvxC as compared to ShamC, and in OvxC + E2 it is also found at mid-range, but this time not significantly different from OvxC. C20:5n-3, C22:5n-3, C22:6n-3, C20:4n-6 and C22:4n-6 all show the same profile: increased in OvxC but only partially restored in OvxC + E2. The total amount of unsaturated fatty acids (∑UFA = MUFA + PUFA) and the ratio ∑SFA/∑UFA showed similar profile: significantly different in Ovx when compared to ShamC, and only partial restoration in OvxC + E2.

The RET Mono–Di FA profile of rats fed the control diet is shown in Table 3. There were no differences in SFA amongst the three groups. The ∑MUFA was significantly higher in OvxC in comparison to ShamC, attributed to the higher levels of C18:1n-9 and C20:1n-9 observed in OvxC. Interestingly, the differences found between OvxC + E2 and ShamC were not significantly different.

Both n-3 and n-6 families were overall reduced in OvxC in comparison with ShamC, with only C22:5n-3 and C22:6n-3 reaching statistically significant reductions. Although statistically similar, ∑n-3 was 19% lower in OvxC, and ∑n-6 16% lower, as compared to ShamC. Following the same pattern described in the CE fraction above, OvxC + E2 rats tended to show a normalization of their Mono–Di FA levels.

### Changes in monoglyceride, diglyceride and cholesteryl ester FA profile induced by the interaction of ovariectomy and lard diet are partially restored by oestrogen replacement therapy

The FA composition of RET CE and Mono–Di fractions from rats fed the lard-enriched diet are presented in Tables 4 and 5. Regarding the FA composition of CE (Table 4), no statistically significant differences were found in SFA, except for C14:0, which was found increased in OvxL as compared to ShamL, and decreased in OvxL + E2. An identical pattern was found in the MUFA family, in which no differences was found, except for C14:1n-7. Interestingly, the differences found in the CE PUFAs of the control diet-fed group, described in Table 2, were no longer observed in the lard diet-fed groups; no statistically significant differences were found in the n-3 or n-6 families, nor in ∑PUFA, ∑UFA or the ratio ∑SFA/∑UFA.

**Table 4:** Fatty acid profile of RET cholesteryl ester lipids obtained from ovariectomized (Ovx) or Sham operated rats, fed a lard-enriched (L) diet, subjected or not to oestrogen replacement therapy (E2).

Fatty acid		% of total fatty acids		
		ShamL	OvxL	OvxL + E2
C14:0	Myristic acid	0.36 ± 0.09	0.56 ± 0.01*	0.27 ± 0.01^#^
C16:0	Palmitic acid	13.38 ± 3.81	13.65 ± 1.44	11.80 ± 1.81
C18:0	Stearic acid	3.49 ± 1.04	3.77 ± 1.19	3.14 ± 0.51
C20:0	Arachidic acid	0.04 ± 0.02	0.02 ± 0.03	0.03 ± 0.01
C24:0	Behenic acid	1.77 ± 1.73	1.01 ± 0.44	0.74 ± 0.45
∑ SFA		19.04 ± 4.33	19.01 ± 2.18	15.99 ± 2.05
C14:1n-7	Myristoleic acid	0.01 ± 0.01	0.03 ± 0.01*	0.01 ± 0.01^#^
C16:1n-7	Palmitoleic acid	1.67 ± 0.37	1.77 ± 0.32	1.46 ± 0.14
C18:1n-9	Oleic acid	29.10 ± 6.92	26.04 ± 4.22	30.32 ± 3.02
C18:1n-7	cis-vaccenic acid	2.39 ± 0.34	2.22 ± 0.19	2.09 ± 0.29
C20:1n-9	Eicosenoic acid	0.14 ± 0.08	0.09 ± 0.05	0.15 ± 0.06
C18/C18:1		0.11 ± 0.02	0.14 ± 0.05	0.10 ± 0.01
C16:0/C16:1n-7		8.82 ± 4.92	7.81 ± 0.60	8.10 ± 1.02
∑ MUFA		33.30 ± 6.98	30.14 ± 4.73	33.87 ± 2.95
C18:3n-3	Alpha-linolenic acid (ALA)	4.07 ± 2.19	4.23 ± 0.82	3.79 ± 0.60
C20:5n-3	Eicosapentaenoic acid (EPA)	0.04 ± 0.02	0.04 ± 0.02	0.04 ± 0.01
C22:5n-3	Docosapentaenoic acid (DPA)	0.13 ± 0.06	0.15 ± 0.05	0.15 ± 0.07
C22:6n-3	Docosahexaenoic acid (DHA)	0.10 ± 0.05	0.07 ± 0.06	0.14 ± 0.06
∑ n-3		4.36 ± 2.28	4.51 ± 0.84	4.13 ± 0.67
C18:2n-6	Linoleic acid (LA)	40.31 ± 8.01	43.76 ± 5.59	43.23 ± 4.33
C18:3n-6	Gamma-linoleic acid	0.10 ± 0.06	0.11 ± 0.02	0.10 ± 0.03
C20:2n-6	Eicosadienoic acid (EDA)	0.28 ± 0.07	0.21 ± 0.09	0.30 ± 0.05
C20:3n-6	Dihomo-gamma linoleic acid	0.23 ± 0.11	0.22 ± 0.02	0.25 ± 0.05
C20:4n-6	Arachidonic acid (AA)	0.99 ± 0.52	0.96 ± 0.24	0.98 ± 0.14
C22:4n-6	Docosatetraenoic acid (DTA)	0.17 ± 0.07	0.17 ± 0.04	0.21 ± 0.04
∑ n-6		42.07 ± 8.68	45.43 ± 5.73	45.07 ± 4.46
∑ n-6/∑ n-3		14.75 ± 12.99	10.23 ± 1.21	11.01 ± 0.77
∑ PUFA		46.43 ± 2.4	49.94 ± 3.2	49.20 ± 2.5
∑ UFA		79.74 ± 4.23	80.08 ± 2.38	83.06 ± 2.17
∑ SFA/∑ UFA		0.24 ± 0.07	0.24 ± 0.03	0.19 ± 0.03

Data presented as means ± standard error (SE) of the % of total FAs. n = 6 for each group.

*SFA* saturated fatty acids, *MUFA* monounsaturated fatty acids, *PUFA* polyunsaturated fatty acids.

*p < 0.05 vs ShamL; #p < 0.05 OvxL vs OvxL + E2.

**Table 5:** Fatty acid profile of RET monoglyceride diglyceride lipids obtained from ovariectomized (Ovx) or Sham operated rats, fed a lard-enriched (L) diet, subjected or not to oestrogen replacement therapy (E2).

Fatty acid		% of total fatty acids		
		ShamL	OvxL	OvxL + E2
C14:0	Myristic acid	0.39 ± 0.26	0.82 ± 0.18*	0.30 ± 0.14^#^
C16:0	Palmitic acid	18.76 ± 5.68	24.11 ± 1.54	20.74 ± 1.83
C18:0	Stearic acid	5.46 ± 1.73	7.64 ± 0.65*	6.75 ± 0.59
C20:0	Arachidic acid	0.04 ± 0.03	0.06 ± 0.02	0.05 ± 0.02
∑ SFA		24.99 ± 7.37	32.95 ± 1.98*	28.24 ± 2.01
C14:1n-7	Myristoleic acid	0.01 ± 0.01	0.03 ± 0.02	0.01 ± 0.01
C16:1n-7	Palmitoleic acid	1.66 ± 0.36	1.94 ± 0.28	1.34 ± 0.11^#^
C18:1n-9	Oleic acid	32.46 ± 5.50	34.18 ± 4.67	37.64 ± 1.87
C18:1n-7	cis-vaccenic acid	2.51 ± 0.38	2.48 ± 0.16	2.24 ± 0.16
C20:1n-9	Eicosenoic acid	0.22 ± 0.09	0.14 ± 0.08	0.27 ± 0.02^#^
C18/C18:1		0.15 ± 0.04	0.21 ± 0.04*	0.17 ± 0.02
C16:0/C16:1n-7		11.36 ± 2.93	12.59 ± 1.48	15.55 ± 1.56*
∑ MUFA		37.14 ± 5.87	39.20 ± 4.76	41.83 ± 1.80
C18:3n-3	Alpha-linolenic acid (ALA)	1.98 ± 1.62	1.15 ± 0.31	1.07 ± 0.21
C20:5n-3	Eicosapentaenoic acid (EPA)	0.02 ± 0.01	0.06 ± 0.05	0.01 ± 0.01
C22:5n-3	Docosapentaenoic acid (DPA)	0.08 ± 0.07	0.09 ± 0.07	0.05 ± 0.03
C22:6n-3	Docosahexaenoic acid (DHA)	0.05 ± 0.06	0.12 ± 0.11	0.02 ± 0.01
∑ n-3		2.13 ± 1.75	1.41 ± 0.48	1.15 ± 0.24
C18:2n-6	Linoleic acid (LA)	28.46 ± 11.17	21.26 ± 2.94	24.06 ± 1.65
C18:3n-6	Gamma-linoleic acid	0.06 ± 0.04	0.04 ± 0.02	0.04 ± 0.04
C20:2n-6	Eicosadienoic acid (EDA)	0.25 ± 0.06	0.12 ± 0.07*	0.24 ± 0.06^#^
C20:3n-6	Dihomo-gamma linoleic acid	0.39 ± 0.14	0.36 ± 0.20	0.25 ± 0.11
C20:4n-6	Arachidonic acid (AA)	0.51 ± 0.31	0.30 ± 0.14	0.41 ± 0.14
C22:4n-6	Docosatetraenoic acid (DTA)	0.10 ± 0.09	0.04 ± 0.02	0.09 ± 0.04
∑ n-6		29.76 ± 11.52	22.05 ± 3.16	25.09 ± 1.85
∑ n-6/∑ n-3		16.93 ± 5.56	16.79 ± 4.67	22.36 ± 3.68
∑ PUFA		31.92 ± 2.4	23.46 ± 3.2*	26.24 ± 2.5
∑ UFA		69.03 ± 9.45	62.66 ± 2.80	68.08 ± 1.73
∑ SFA/∑ UFA		0.38 ± 0.15	0.53 ± 0.05	0.42 ± 0.03

Data presented as means ± standard error (SE) of the % of FAs. n = 6 for each group.

*SFA* saturated fatty acids, *MUFA* monounsaturated fatty acids, *PUFA* polyunsaturated fatty acids.

*p < 0.05 vs ShamL; #p < 0.05 OvxL vs OvxL + E2.

The RET Mono–Di FA composition of rats fed the lard diet is shown in Table 5. OvxL rats showed higher ∑SFA as compared to ShamL, result attributed to significantly higher levels of C14:0 and C18:0 in OvxL. C16:0 levels nearly reached statistical significance, with a *p* value of 0.08. Following the same pattern observed in previous results, OvxL + E2 rats showed a partial SFA restoration.

Within the MUFA family, the ∑MUFA content was similar across the three groups, although the levels of two FA appear to counteract each other: C16:1n-7 was significantly higher in OvxL as compared to OvxL + E2, whilst C20:1n-9 was significantly lower. The ∑n-3 and ∑n-6 families were statistically similar between ShamL and OvxL, but in both cases a tendency for reduction in OvxL was observed, which was found to be statistically different when both families were summed together as ∑PUFA. For both PUFA families, oestrogen replacement therapy appears to bring FA levels closer to the ShamL group. No differences were observed in ∑UFA or ∑SFA/∑UFA across the three groups.

## Discussion

Menopause is an important risk factor for the development of obesity, which becomes further exacerbated when associated with the consumption of energy dense diets^33^. Our group has recently demonstrated that ovariectomy modified the fatty acid profile of RET total lipid extract, which was marginally normalized by oestrogen replacement^30^. Those results confirm that the loss of ovarian hormones, combined or not with the consumption of a lard-enriched diet, could lead to impaired lipid and fatty acid metabolism in visceral adipose tissue.

In order to further examine our hypothesis, we investigated the fatty acid composition of cholesteryl ester (CE) and monoglyceride diglyceride (Mono–Di) lipid fractions extracted from retroperitoneal white adipose tissue, firstly to determine to what extent lipids are affected by ovarian losses, and secondly to evaluate how efficient oestrogen replacement is at restoring any observed alteration.

As our most recently published results^30^ most likely reflect the predominant triglyceride portion of the RET adipocyte fatty acid composition, in order to further examine our hypothesis, we investigated the fatty acid composition of cholesteryl ester (CE) and monoglyceride diglyceride (Mono–Di) lipid fractions extracted from retroperitoneal white adipose tissue, firstly to determine to what extent these dynamic lipid fractions are affected by ovarian losses, and secondly to evaluate how efficient oestrogen replacement is at restoring any observed alteration.

Ovariectomy alone increased body weight gain and adiposity due to increased feed efficiency, as food intake was not increased (Table 1). Insulin and leptin levels, leptin/adiponectin ratio, and HOMA indexes also increased after ovariectomy. The observed alterations were further exacerbated by high-fat diet ingestion. Our findings are consistent with the demonstration that impaired glucose homeostasis influences adipose tissue inflammation during high-fat intake^34^. In our study, whilst oestradiol replacement was able to attenuate the serum parameters altered by ovariectomy alone, the same was not observed when in combination with high-fat diet ingestion. We suggest that due to the much poorer metabolic background of the lard diet-fed ovariectomized rats in comparison to the control diet-fed ovariectomized rats, the present oestradiol replacement therapy of 2.8 µg/day was possibly not sufficient to induce positive metabolic effects. Our suggestion agrees with a similar experiment in ovariectomized mice fed a lard-enriched diet, in which an oestradiol dose of 1.7 µg/day protected the mice from insulin resistance^35^. The oestrogen dose to mice in the study of Riant was relatively much higher in comparison to our study in rats, although the oestrogen replacement dose chosen for our study appears to be compatible with the human dosage of the average transdermal replacement therapy commonly used for postmenopausal women^36,37^.

The CE FA analyses from rats fed the control diet showed that SFA decreased in OvxC in comparison to ShamC, while both n-3 and n-6 PUFA levels increased and MUFAs remained unchanged (Table 2). However, the CE FA analyses from rats fed the lard diet showed similar SFA, MUFAs and PUFAs levels, with no further alterations than the ones caused by ovariectomy alone (Table 4). These results agree with CE being a dynamic fraction and a reflection of global metabolic status, playing a major role as an integral component of membrane lipid rafts and as vehicle for the exportation of FA stored intracellularly in adipocytes^38,39^.

The decreased SFA levels observed in ovariectomized rats, particularly palmitic acid, was previously reported in the visceral adipose tissue of postmenopausal women, where a decreased ratio of saturation/unsaturation was identified^40^. Whilst the study of Garaulet^40^ suggests that dietary factors combined with the age of subjects may play an important role in this phenomenon, our results suggest that ovariectomy alone may have had an effect, once SFA content decreased from 30.37% in ShamC down to 19.15% in OvxC, whilst ShamL showed 19.04% SFA, a content similar to 19.01% in OvxL (Table 2).

Decreased levels of SFA in serum phospholipids in postmenopausal women were observed previously^41^, whilst another report showed increased PUFA levels in plasma CE in postmenopausal women, alongside a positive association with adiposity^42^. Our findings that CE PUFAs were significantly higher in OvxC as compared to ShamC suggest the mobilization of intracellularly stored PUFA for utilization elsewhere, with CE deployed as a vehicle for the exportation of essential EFAs from within the cell. Our hypothesis is further corroborated by the Mono–Di findings shown in Table 3, in which DPA and DHA are significantly reduced in OvxC as compared to ShamC, and although not statistically significant, the ∑PUFA was 15.7% lower in OvxC (31.07% ∑PUFA) versus ShamC (36.87% ∑PUFA). We suggest that, in ovariectomised rats, adipocytes are exporting PUFAs and retaining SFAs.

The changes in CE and Mono–Di FA observed in the lard fed group were not identical to the changes observed in the control group. Whilst there was a tendency for increased ∑PUFA in CE of OvxL (49.94% in OvxL *versus* 46.43% in ShamL) (Table 4), such differences did not reach the statistical significance observed between OvxC *versus* ShamC (Table 2). Nonetheless, the Mono–Di ∑PUFA content was significantly lower in OvxL (23.46%) as compared to ShamL (31.92%) (Table 5).

The lard-fed rats in the present study were exposed to an EFA-deficient diet. The deleterious impact of saturated fat-rich diets upon peripheral tissue fatty acid profile has been previously demonstrated by us^43^ and others^44,45^. In a chronically deficient EFA diet, it may be possible that the biochemically harsh conditions prevented the RET CE from further adapting in ovariectomy, but it appears the Mono–Di fraction, which is located intracellularly in abundance, was able to buffer some of those unfavourable conditions, confirming the altruist role of the adipose tissue in protecting the body^46^.

Oestrogen replacement was able to partially attenuate the CE FA alterations observed in ovariectomy, in which it appears to return the levels of some FA, including behenic, AA, EPA and DHA levels, to levels similar to those found in Sham rats. It has been documented that hormone replacement therapy was able to decrease the levels of behenic acid in serum phospholipids^41^ as well as decreased AA levels in whole blood of postmenopausal women^47^. In the particular case of AA, we acknowledge the disagreement of our findings with previous studies showing that hormone replacement increase AA levels, mostly due to alterations in Δ6-desaturase activity caused by oestrogen^41,48^. It is worth noting however that whilst those results refer to whole blood, in which there is transport of EFAs by phospholipids for the brain and other prime tissues, in our study the results were observed in retroperitoneal white adipose tissue neutral lipid species.

As compared to ShamC, the OvxC group showed in the Mono–Di fraction no differences in SFA, but the ∑MUFA was significantly increased in OvxC, mainly attributed to higher oleic acid levels (Table 3). The stearoyl-CoA desaturase, also known as Δ9-desaturase, is the intracellular enzyme that catalyses the rate-limiting conversion of palmitoyl-CoA and stearoyl-CoA to palmitoleic and oleic acids^49^. Oleic acid is the predominant FA stored in triglycerides of adipose tissue^50^, with the formation of oleic acid being a direct product of Δ9 desaturase activity on fatty acyl-CoA substrates^51^. Alessandri and colleagues^52^ reported increased Δ9-desaturase levels following ovariectomy in rats, whilst oestrogen has been shown to suppress its expression in liver and adipose tissue^53,54^. Overall, such findings may explain the increased oleic acid levels in Mono–Di following ovariectomy, with subsequent tendency for reduction following oestrogen replacement.

DPA and DHA were significantly decreased in Mono–Di OvxC (Table 3), and the reduced levels of the n-6 counterparts AA and docosatetraenoic (DTA) did not reach statistically significant differences, even though a tendency was clear. Our findings that the Mono–Di fraction contained more MUFA and less PUFA agree with previous reports showing that Mono–Di molecules have an important role as intracellular storage^55,56^, and, as opposed to CE, Mono–Di are relatively more inert. The alternate increase and decrease of PUFA levels in CE and Mono–Di respectively may suggest the transfer of PUFA from within the cell to CE for membrane utilization and exportation, resulting in Mono–Di left with higher MUFAs.

Our suggestion of increased PUFA exportation through CE, but not SFA or MUFA, in OvxC rats may be attributed to the ability of peripheral tissues to synthesise SFA and MUFA but not essential PUFA. Interestingly, Belkaid and colleagues reported that 17β-oestradiol induces stearoyl-CoA desaturase-1 expression in oestrogen-responding cancer cells^57^, whilst in the opposite direction, Alessandri and colleagues^52^ reported increased hepatic Δ9-desaturase levels following ovariectomy in rats. In our study, the ratio C18/C18:1 was significantly decreased in CE of OvxC as compared to ShamC (Table 2), and significantly increased in the Mono–Di fraction of OvxL *versus* ShamL (Table 3). We also found more MUFA in Mono–Di in OvxC as compared to ShamC, but no MUFA changes in CE between OvxC and ShamC. We have not measured stearoyl-CoA desaturase activity in our study; however, as a higher C18/C18:1 ratio denotes proportionally more saturated than monounsaturated species, we further speculate that there is transfer of fatty acids from one compartment to another.

The association of lard diet with ovariectomy (OvxL) did not trigger differences in MUFA content in the Mono–Di fraction; however, it significantly increased ∑SFA and decreased ∑PUFA in relation to ShamL (Table 5). Differently from OvxC, the ∑SFA increase in OvxL may be traced to an effect of the diet alone. Our view is corroborated by a report on rats that showed a high-fat diet regimen containing 60% of kcal from fat increased the content of diglycerides in the liver, associated with systemic insulin resistance^58^. Additionally, it has been shown that a regimen of n-3 FA supplementation improved insulin sensitivity and decreased the content of diglycerides in visceral adipose tissue of rats fed a high-fat diet^59^.

OvxC + E2 showed partial restoration of ∑MUFA levels in Mono–Di, whist showing little effect in PUFA levels. Interestingly, ∑SFA and ∑PUFA were partially restored in Mono–Di in OvxL + E2. We believe oestrogen replacement may be responsible for partial attenuation of the changes observed in ovariectomy, alone or in combination with a lard diet. Our results agree with previous findings that oestrogen replacement ameliorated the overall lipid metabolism in ovariectomy alone^60^ as well as in association of ovariectomy with a high-fat diet^61^.

The limitation of this study was to not include triglycerides, which represents a predominant fraction of neutral lipids within adipose tissue. Our choice to exclude triglycerides was based on data from our previous study where we demonstrated that ovariectomy leads to a disruption of the fatty acid composition of total lipid extract, which includes, in its majority, the triglyceride portion of neutral lipids^30^. By choosing to focus on cholesteryl esters, monoglycerides and diglycerides we aimed to investigate the more subtle and dynamic portions of the neutral lipid synthesis pathway within the adipose tissue that would confirm any metabolic changes taking place. Nevertheless, we acknowledge that including the triglycerides in this study could add to further its relevance, and deserves attention for future studies.

In conclusion, the present study showed that ovariectomy induced dramatic changes in the cholesteryl ester and monoglyceride/diglyceride fatty acid profiles of retroperitoneal white adipose tissue. Such changes appear to have been more dramatic in rats that received the control diet, as compared to the rats that received a diet enriched with lard. The control diet-fed rats may have shown greater capacity to adapt to hormone deficiency possibly due to a better fatty acid profile and a lower mild chronic pro-inflammatory background, as compared to the rats that chronically received the lard diet. We have also found that hormone replacement therapy tended to restore the level of some of the fatty acid families, but such findings were not consistently significant across all lipid families analysed in the present study. We speculate that hormone replacement therapy alone may not be sufficient to restore fatty acid profile changes observed in ovariectomy. Further studies are necessary to investigate whether hormone replacement therapy combined with positive nutrition interventions could promote better outcomes for menopaused women, particularly those subjected to nutrient-deficient diets.

## Material and methods

### Experimental procedures

All experiments were conducted in accordance with the Committee in Research Ethics of the Universidade Federal de São Paulo (CEUA no.: 2172030315/2016), through the guidelines of the Conselho Nacional de Controle de Experimentação Animal (CONCEA), Ministry of Science and Technology, Brazil. Detailed experimental procedures, body composition analyses and biochemical measurements adopted in the present study have been published previously^6^.

Briefly, twelve-week-old female Wistar rats were either ovariectomized (Ovx, n = 24) or sham operated (Sham, n = 12) under ketamine/xylazine anaesthesia (66/33 mg/kg intraperitoneally). From the total of 24 Ovx rats, twelve received 17β-oestradiol replacement (Ovx + E2 group) through the insertion of subcutaneous pellets (0.25 mg/pellet, 90-day release, Innovative Research of America, Sarasota, Florida, USA). Penicillin (60.000U intramuscularly) and ibuprofen (1 mg/kg BW orally) were administered for two days following surgery.

Rats were maintained under controlled 12 h light/dark cycle (lights on at 6:00 am) and temperature (23 ± 1 °C) with *ab libitum* food and water for 12 weeks. Upon housing, the three above groups were randomly sub-divided six by six, according to the diet offered. ShamC, OvxC and OvxC + E2 received standard rat chow (2.87 kcal/g, 15% of energy from fat, Nuvilab CR-1, Nuvital Nutrientes SA, Colombo, PR, Brazil). The ShamL, OvxL and OvxL + E2 groups received a high-fat lard-enriched diet (3.60 kcal/g, 45% energy from fat).

The lard-enriched diet was prepared by adding to the powdered standard chow 18% lard (w/w) (Cooperativa Central Aurora de Alimentos, Chapecó, Santa Catarina, Brazil), 2% soybean oil (w/w), 10% sucrose (w/w), 20% casein (w/w) to obtain the protein content of the control diet, and 0.02% (w/w) butylated hydroxytoluene (BHT). The mixture was mechanically mixed with lukewarm water for thorough homogenization of all ingredients, passed through a milling machine to produce pellets and dried in a forced ventilation oven for 24 h at 60 °C. The diet was stored at -20 °C and offered in standardized portions. The leftovers were weighted and discarded. Body weight and 24 h food mass intake were measured weekly. Feed efficiency was calculated as (body weight gain / energy intake) × 100.

Twelve weeks after surgery, rats were fasted for 24 h and sacrificed by decapitation under thiopental anaesthesia (80 mg/kg intraperitoneally). Trunk blood was collected. RET were dissected, weighed, snap-frozen in liquid nitrogen and stored at – 80 ºC. The uteri were weighed for confirmation of completeness of ovariectomy.

### Bodily measurements and serum biomarkers

Initial and final body weight, uterus and RET mass, serum leptin, adiponectin, glucose, insulin, total cholesterol, HDL cholesterol and triglycerides were quantified. The HOMA index was calculated as previously described^6^. The sensitivity, intra-assay and inter-assay variations of the ELISA kits used to determine the serum levels of leptin, insulin and adiponectin were, respectively, 0.08 ng/mL; 2.49% and 3.93% for leptin; 0.1 ng/mL; 1.33% and 6.71% for insulin; 0.4 ng/mL; 1.18% and 7.34% for adiponectin.

### RET lipid extraction, solid-phase chromatographic separation and fatty acid analysis

Aliquots of 1000 mg of RET was homogenized and extracted in hexane/isopropanol (3:2 v/v) containing 0.01% BHT. After addition of chloroform/methanol/water (2:1:1 v/v/v), the samples were centrifuged at 10,000*g* for 10 min. The organic layer was separated and evaporated to complete dryness with oxygen-free nitrogen (OFN). The lipids were partitioned again in chloroform/methanol/water (8:4:3 v/v/v), the chloroform layer was obtained, dried under OFN and kept in airtight glass vials under OFN at – 20 ºC.

CE and Mono–Di fractions were chromatographically obtained according to a previously established protocol^31^. Briefly, sample lipid residues were extracted twice in succession with 0.5 ml of isooctane-ethyl acetate (80:1 v/v) and applied to previously prepared silica gel columns (Thermo Scientific HyperSep silica columns; 100 mg bed weight) gravimetrically. CE were eluted with 5 ml of isooctane-ethyl acetate (20:1 v/v), followed by the Mono–Di fraction elution with 4.5 ml of isooctane-ethyl acetate (75:25 v/v). The collected fractions were immediately dried under OFN and kept in airtight glass vials under OFN at – 20 ºC until derivatization.

FA analysis was performed as previously described by our group^32^. Briefly, fatty acid methyl esters (FAMEs) were obtained by heating lipid samples in sealed glass tubes at 70 ºC for 3 h with 15% acetyl chloride in dry methanol under OFN. The reaction was stopped with 5% NaCl solution at room temperature and FAMEs were extracted with three washes of petroleum spirit containing 0.01% BHT. Extracted FAMEs were analysed by gas chromatography with flame ionization detector (GC2010 Plus, Shimadzu, Kyoto, Japan) equipped with a Trace TR-FAME capillary column (60 m × 0.32 mm × 0.25 µm, Thermo Scientific, Rockford, IL, USA). FAMEs were identified by comparison with the retention times of previously injected authentic standards (Sigma-Aldrich, United Kingdom). Peak areas were analysed using Shimadzu software LabSolutions (Shimadzu, Kyoto, Japan).

### Statistical analyses

Body weight, RET mass and serum parameters were tested for normality (Shapiro–Wilk test) and homoscedasticity (Levene’s test). Normally distributed variables (means ± standard error of the mean) were analysed by two-way ANOVA and Tukey post hoc test. Non-parametric variables (median and interquartile range) were analysed by Kruskal–Wallis followed by multiple comparisons. Tests were performed with Statistica 12 Software (StatSoft, Tulsa, OK, USA).

CE and Mono–Di FA composition results were compared by one-way ANOVA, with groups separated according to their diet. Tests were performed by SPSS software (IBM, Chicago, IL, USA). Fatty acid results are presented as mean and standard deviation of the mean, and the level of statistical significance was set at p < 0.05.

## References

[1] Donato GB, Fuchs SC, Oppermann K, Bastos C, Spritzer PM (2006). Association between menopause status and central adiposity measured at different cutoffs of waist circumference and waist-to-hip ratio. Menopause.

[2] Horber FF, Gruber B, Thomi F (1997). Effect of sex and age on bone mass, body composition and fuel metabolism in humans. Nutrition.

[3] Dornellas APS, Boldarine VT, Pedroso AP (2018). High-fat feeding improves anxiety-type behavior induced by ovariectomy in rats. Front. Neurosci..

[4] Rettberg JR, Yao J, Brinton RD (2014). Oestrogen: A master regulator of bioenergetic systems in the brain and body. Front. Neuroendocrinol..

[5] Ren H, Liang D, Shen G (2016). Effects of combined ovariectomy with dexamethasone on rat lumbar vertebrae. Menopause.

[6] Boldarine VT, Pedroso AP, Neto NIP (2019). High-fat diet intake induces depressive-like behavior in ovariectomized rats. Sci. Rep..

[7] Cusi K (2010). The role of adipose tissue and lipotoxicity in the pathogenesis of type 2 diabetes. Curr. Diab. Rep..

[8] Yamatani H, Takahashi K, Yoshida T, Takata K, Kurachi H (2013). Association of oestrogen with glucocorticoid levels in visceral fat in postmenopausal women. Menopause.

[9] Robichaud PP, Surette ME (2015). Polyunsaturated fatty acid-phospholipid remodeling and inflammation. Curr. Opin. Endocrinol. Diabetes Obes..

[10] Bueno AA, Oyama LM, de Oliveira C (2008). Effects of different fatty acids and dietary lipids on adiponectin gene expression in 3T3-L1 cells and C57BL/6J mice adipose tissue. Pflugers Arch..

[11] Galic S, Oakhill JS, Steinberg GR (2010). Adipose tissue as an endocrine organ. Mol. Cell Endocrinol..

[12] Karastergiou K, Mohamed-Ali V (2010). The autocrine and paracrine roles of adipokines. Mol. Cell Endocrinol..

[13] Makki K, Froguel P, Wolowczuk I (2013). Adipose tissue in obesity-related inflammation and insulin resistance: Cells, cytokines, and chemokines. ISRN Inflamm..

[14] Ahima RS (2006). Adipose tissue as an endocrine organ. Obesity (Silver Spring).

[15] Lee TH, Cheng KK, Hoo RL, Siu PM, Yau SY (2019). The novel perspectives of adipokines on brain health. Int. J. Mol. Sci..

[16] Deslypere JP, Verdonck L, Vermeulen A (1985). Fat tissue: A steroid reservoir and site of steroid metabolism. J. Clin. Endocrinol. Metab..

[17] Barakat R, Oakley O, Kim H, Jin J, Ko CJ (2016). Extra-gonadal sites of estrogen biosynthesis and function. BMB Rep..

[18] Cerqueira NM, Oliveira EF, Gesto DS (2016). Cholesterol biosynthesis: A mechanistic overview. Biochemistry.

[19] Bracht, J.R., Vieira‐Potter, V.J., De Souza Santos, R. *et al*. The role of estrogens in the adipose tissue milieu. *Ann. N. Y. Acad. Sci.* **1461**(1), 127–143 (2020). 10.1111/nyas.14281 31868931

[20] Goldstein JL, DeBose-Boyd RA, Brown MS (2006). Protein sensors for membrane sterols. Cell.

[21] Zee P (1968). Lipid metabolism in the newborn II. Neutral lipids. Pediatrics.

[22] Hamilton JG, Comai K (1984). Separation of neutral lipids and free fatty acids by high-performance liquid chromatography using low wavelength ultraviolet detection. J. Lipid Res..

[23] Moilanen T, Nikkari T, Räsänen L (1983). Plasma cholesteryl ester fatty acids in 3- and 12-year-old Finnish children. Atherosclerosis.

[24] Sandker GW, Kromhout D, Aravanis C (1993). Serum cholesteryl ester fatty acids and their relation with serum lipids in elderly men in Crete and The Netherlands. Eur. J. Clin. Nutr..

[25] Sanders K, Johnson L, O'Dea K, Sinclair AJ (1994). The effect of dietary fat level and quality on plasma lipoprotein lipids and plasma fatty acids in normocholesterolemic subjects. Lipids.

[26] Pike LJ (2003). Lipid rafts: Bringing order to chaos. J. Lipid Res..

[27] Simons K, Sampaio JL (2011). Membrane organization and lipid rafts. Cold Spring Harb. Perspect. Biol..

[28] Athenstaedt K, Daum G (2006). The life cycle of neutral lipids: Synthesis, storage and degradation. Cell Mol. Life Sci..

[29] Pereira-Dutra FS, Teixeira L, de Souza Costa MF, Bozza PT (2019). Fat, fight, and beyond: The multiple roles of lipid droplets in infections and inflammation. J. Leukoc. Biol..

[30] Boldarine VT, Pedroso AP, Caroline BT (2020). Ovariectomy modifies lipid metabolism of retroperitoneal white fat in rats: A proteomic approach. Am. J. Physiol. Endocrinol. Metab..

[31] Ingalls ST, Kriaris MS, Xu Y (1993). Method for isolation of non-esterified fatty acids and several other classes of plasma lipids by column chromatography on silica gel. J. Chromatogr. B Biomed. Sci. Appl..

[32] Bueno AA, Brand A, Neville MM (2015). Erythrocyte phospholipid molecular species and fatty acids of Down syndrome children compared with non-affected siblings. Br. J. Nutr..

[33] Everson-Rose SA, Lewis TT, Karavolos K (2009). Depressive symptoms and increased visceral fat in middle-aged women. Psychosom. Med..

[34] Vatarescu M, Bechor S, Haim Y (2017). Adipose tissue supports normalization of macrophage and liver lipid handling in obesity reversal. J. Endocrinol..

[35] Riant E, Waget A, Cogo H (2009). Oestrogens protect against high-fat diet-induced insulin resistance and glucose intolerance in mice. Endocrinology.

[36] Hill DA, Crider M, Hill SR (2016). Hormone therapy and other treatments for symptoms of menopause. Am. Fam. Phys..

[37] Nair AB, Jacob S (2016). A simple practice guide for dose conversion between animals and human. J. Basic Clin. Pharm..

[38] Krause BR, Hartman AD (1984). Adipose tissue and cholesterol metabolism. J. Lipid Res..

[39] Sezgin E, Levental I, Mayor S, Eggeling C (2017). The mystery of membrane organization: Composition, regulation and roles of lipid rafts. Nat. Rev. Mol. Cell Biol..

[40] Garaulet M, Pérez-Llamas F, Zamora S, Tébar FJ (2002). Estudio comparativo del tipo de obesidad en mujeres pre y posmenopáusicas: relación con el tamaño adipocitario, la composición de la grasa y diferentes variables endocrinas, metabólicas, nutricionales y psicológicas (Comparative study of the type of obesity in pre- and postmenopausal women: Relationship with fat cell data, fatty acid composition and endocrine, metabolic, nutritional and psychological variables). Med. Clin. (Barc).

[41] Stark KD, Park EJ, Holub BJ (2003). Fatty acid composition of serum phospholipid of premenopausal women and postmenopausal women receiving and not receiving hormone replacement therapy. Menopause.

[42] Lewis-Barned NJ, Sutherland WH, Walker RJ (2000). Plasma cholesteryl ester fatty acid composition, insulin sensitivity, the menopause and hormone replacement therapy. J. Endocrinol..

[43] Dornellas AP, Watanabe RL, Pimentel GD (2015). Deleterious effects of lard-enriched diet on tissues fatty acids composition and hypothalamic insulin actions. Prostaglandins Leukot. Essent. Fatty Acids.

[44] Brufau G, Canela MA, Rafecas M (2006). A high-saturated fat diet enriched with phytosterol and pectin affects the fatty acid profile in guinea pigs. Lipids.

[45] Tranchida F, Tchiakpe L, Rakotoniaina Z (2012). Long-term high fructose and saturated fat diet affects plasma fatty acid profile in rats. J. Zhejiang Univ. Sci. B.

[46] Flatt JP (1970). Energy metabolism and the control of lipogenesis in adipose tissue. Horm. Metab. Res..

[47] Cybulska AM, Skonieczna-Żydecka K, Drozd A (2019). Fatty acid profile of postmenopausal women receiving, and not receiving, hormone replacement therapy. Int. J. Environ. Res. Public Health.

[48] Piperi C, Tzivras M, Kalofoutis C (2005). Effects of hormone replacement therapy on the main fatty acids of serum and phospholipids of postmenopausal women. In Vivo.

[49] Paton CM, Ntambi JM (2009). Biochemical and physiological function of stearoyl-CoA desaturase. Am. J. Physiol. Endocrinol. Metab..

[50] Jeffcoat R (2007). Obesity - A perspective based on the biochemical interrelationship of lipids and carbohydrates. Med. Hypotheses.

[51] Flowers MT, Ntambi JM (2008). Role of stearoyl-coenzyme A desaturase in regulating lipid metabolism. Curr. Opin. Lipidol..

[52] Alessandri JM, Extier A, Al-Gubory KH (2011). Ovariectomy and 17β-estradiol alter transcription of lipid metabolism genes and proportions of neo-formed n-3 and n-6 long-chain polyunsaturated fatty acids differently in brain and liver. J. Nutr. Biochem..

[53] Paquette A, Wang D, Jankowski M, Gutkowska J, Lavoie JM (2008). Effects of ovariectomy on PPAR alpha, SREBP-1c, and SCD-1 gene expression in the rat liver. Menopause.

[54] Gao H, Bryzgalova G, Hedman E (2006). Long-term administration of estradiol decreases expression of hepatic lipogenic genes and improves insulin sensitivity in ob/ob mice: A possible mechanism is through direct regulation of signal transducer and activator of transcription 3. Mol. Endocrinol..

[55] Ridgway ND, Byers DM, Cook HW, Storey MK (1999). Integration of phospholipid and sterol metabolism in mammalian cells. Prog. Lipid Res..

[56] Baker RC, Nikitina Y, Subauste AR (2014). Analysis of adipose tissue lipid using mass spectrometry. Methods Enzymol..

[57] Belkaid A, Duguay SR, Ouellette RJ, Surette ME (2015). 17β-estradiol induces stearoyl-CoA desaturase-1 expression in estrogen receptor-positive breast cancer cells. BMC Cancer.

[58] Zabielski P, Hady HR, Chacinska M (2015). The effect of high fat diet and metformin treatment on liver lipids accumulation and their impact on insulin action. Sci. Rep..

[59] Chacińska M, Zabielski P, Książek M (2019). The impact of OMEGA-3 fatty acids supplementation on insulin resistance and content of adipocytokines and biologically active lipids in adipose tissue of high-fat diet fed rats. Nutrients.

[60] Babaei P, Mehdizadeh R, Ansar MM, Damirchi A (2010). Effects of ovariectomy and oestrogen replacement therapy on visceral adipose tissue and serum adiponectin levels in rats. Menopause Int..

[61] Buniam J, Chukijrungroat N, Khamphaya T (2019). Oestrogen and voluntary exercise attenuate cardiometabolic syndrome and hepatic steatosis in ovariectomized rats fed a high-fat high-fructose diet. Am. J. Physiol. Endocrinol. Metab..

